# Epilepsy and the “dark” literature of the Greek novelist Demosthenes Voutyras: an outstanding epileptic personality

**DOI:** 10.1055/s-0042-1758443

**Published:** 2023-02-28

**Authors:** Panayiotis Patrikelis, Lambros Messinis, Vasiliki Folia, Grigorios Nasios, Athanasia Alexoudi, Anastasia Verentzioti, Stefanos Korfias, Vasileios Kimiskidis, Stylianos Gatzonis

**Affiliations:** 1 National and Kapodistrian University of Athens, School of Medicine, Evangelismos Hospital, First Department of Neurosurgery, Athens, Greece.; 2 Aristotle University of Thessaloniki, Faculty of Philosophy, School of Psychology, Thessaloniki, Greece.; 3 University Hospital of Patras, School of Medicine, Departments of Neurology and Psychiatry, Patras, Greece.; 4 University of Ioannina, School of Health Sciences, Department of Speech and Language Therapy, Ioannina, Greece.; 5 Aristotle University of Thessaloniki, Faculty of Health Sciences, School of Medicine, 1st Department of Neurology, Thessaloniki, Greece.

**Keywords:** Epilepsy, Seizures, Metaphor, Medicine in Literature, Research Report, Epilepsia, Convulsões, Metáfora, Medicina na Literatura, Relatório de Pesquisa

## Abstract

**Background** The role of temporal lobe epilepsy (TLE) in determining personality traits and neurobehavioral symptoms, collectively known as the interictal behavioral syndrome (also known as Geschwind syndrome or “Gastaut-Geschwind syndrome”), as well as the syndrome's association with the particular artistic expression of many epileptic litterateurs are well known in neurology and psychiatry. A deepening of emotionality along with a serious, highly ethical, and spiritual behavior have been described as positive personality changes among patients with chronic mesial-TLE.

**Objectives** Our narrative-based clinical hypothesis aims at contributing to the ongoing debate on the association between TLE and artistic expression, as well as the latter's supposed implication for epileptology in general and the neuropsychology of epilepsy in particular.

**Methods** Through an analysis of the biography, language, and literary work of Greek novelist Demosthenes Voutyras, we hypothesize that his mystical and dark writing style could be attributed to medial temporal interictal dynamics.

**Conclusions** We suggest that the psycholiterary profile of Voutyras is consistent with the idiosyncratic characteristics of the temporal lobe personality, while a non-dominant temporal lobe contribution has been proposed.

## Resumo

**Antecedentes** O papel da epilepsia do lobo temporal (ELT) na determinação de traços de personalidade e sintomas neurocomportamentais, coletivamente conhecidos como síndrome comportamental interictal (também conhecida como síndrome de Geschwind ou “síndrome de Gastaut-Geschwind”), bem como a associação da síndrome com o expressão de muitos literatos epilépticos são bem conhecidos em neurologia e psiquiatria. Um aprofundamento da emotividade juntamente com um comportamento sério, altamente ético e espiritual tem sido descrito como mudanças positivas de personalidade em pacientes com ELT mesial crônica.

**Objetivos** A nossa hipótese clínica narrativa visa contribuir para o debate em curso sobre a associação entre ELT e a expressão artística, bem como a suposta implicação desta última para a epilepsia em geral e a neuropsicologia da epilepsia em particular.

**Métodos** Através de uma análise da biografia, linguagem e obra literária do romancista grego Demóstenes Voutyras, levantamos a hipótese de que seu estilo de escrita místico e sombrio poderia ser atribuído à dinâmica interictal temporal medial.

**Conclusões** Sugerimos que o perfil psicoliterário de Voutyras é consistente com as características idiossincráticas da personalidade do lobo temporal, enquanto uma contribuição do lobo temporal não dominante foi proposta.

## LIFE AND WORK

Demosthenes Voutyras (1872–1958) is one of the most important Greek writers of the twentieth century, with an imposing work in terms of volume and quality. 1 The writer has direct ties to the city of Messolonghi, due to his mother origins and part of his childhood, which was spent there. Voutyras was born in 1872 on the ship on which his parents traveled to Constantinople, where they stayed for three years. His family then settled in Messolonghi until his father, a few years later, was appointed notary in Piraeus. A severe illness, epilepsy with frequent seizures and an onset in early adolescence, determined the course of his life: it forced him to drop out of high school and become self-taught.

**Figure 1:**
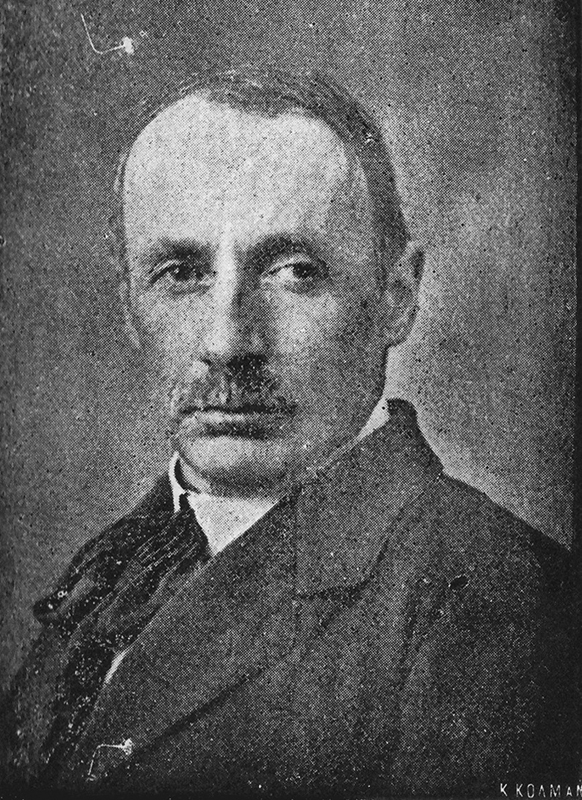
Demosthenes Voutyras (1872–1958).

He started writing as an escape from the devastating consequences of his illness, finding in it a way to express his desires and channel his great inherent vitality. Before devoting himself entirely to literature, Voutyras' father went bankrupt and committed suicide. Thereafter, his possessions were confiscated. Consequently, Voutyras was forced to get a job performing hard manual labor, which had a dramatic impact on him, making him get in touch with the life conditions and mind frame of the people who toil and struggle. The acquired social knowledge in combination with his innate talent transformed him in one of the greatest realistic storytellers in twentieth-century Greece ( Figure 1 ).

Voutyras was awarded the National Prize for Excellence in Letters and Arts (1924) and the Medal of the Municipality of Piraeus (1932). After the end of World War II, his honorary pension was terminated. Furthermore, the Academy of Athens, after two consecutive votes, rejected his application for membership, because Voutyras had supported the left-wing Greek Resistance during the Axis occupation of Greece in 1941–1944. 2

His stories have been translated and published in foreign anthologies and magazines. Many articles by foreign critics 3 have been devoted to his work, and 32 of his books are in circulation, but there is additional yet unpublished material. Voutyras is one of the first litterateurs of the early twentieth century to write a social short story.

Many of his works are also scattered in newspapers and magazines, in which his short stories began to appear in 1901. He published over 30 volumes from 1901 to 1945 ( Table 1 ).

**Table 1:** Volumes of short stories and novels written by Voutyras

English translation	Original titles in modern Greek
***Short stories***	***Διηγήματα***
“Lagas,” 1901	«Λαγκάς», 1901
“Pagan Pope,” 1920	«Παπάς Ειδωλολάτρης», 1920
“Thirty-two tales,” 1921	«Τριάντα δύο διηγήματα», 1921
“Sick life,” 1921	«Ζωή Αρρωστημένη», 1921
“Away from the world,” 1921	«Μακριά απó τον κóσμο», 1921
“The overthrow of the Gods,” 1922	«Το γκρέμισμα των Θεών», 1922
“A dream that does not end,” 1923	«Όνειρο που δεν τελειώνει», 1923
“The mourning of the oxen,” 1923	«Ο θρήνος των βοδιών», 1923
“Persecuted love,” 1923	«Διωγμένη αγάπη», 1923
“Light in the dark,” 1923	«Φως στο σκοτάδι», 1923
“The young Moses,” 1923	«Ο νέος Μωυσής», 1923
“Twenty stories,” 1924	«Είκοσι διηγήματα», 1924
“Aristocratic neighborhood,” 1924	«Αριστοκρατική γειτονιά», 1924
“Food to death,” 1926	«Τροϕή στο θάνατο», 1926
“In the Land of the Wise and the Wild,” 1927	«Στη χώρα των σοϕών και των αγρίων», 1927
“Among the cannibals,” 1928	«Μες στους ανθρωποϕάγους»,1928
“Resurrection of the dead,” 1929	«Ανάσταση νεκρών», 1929
“Animal revolution,” 1929	«Επανάσταση των Ζώων», 1929
“The hen scratching its eye,” 1931	«Η óρνιθα ξύνοντας το μάτι της»,1931
“After a million years,” 1932	«Ύστερα απó εκατομμύρια χρóνια»,1932
“False civilizations,” 1934	«Κάλπικοι πολιτισμοί», 1934
“The Song of the Hanged,” 1935	«Το Τραγούδι του Κρεμασμένου», 1935
“Nights of magic,” 1938	«Νύχτες μαγείας», 1938
“Love in the tombs,” 1943	«Ο έρωτας στους τάϕους», 1943
“Slow dawn,” 1950	«Αργó ξημέρωμα», 1950
***Novels***	***Μυθιστορήματά***
*The vagabonds* , 1921	*Οι Αλανιάρηδες* , 1921
*From Earth to Mars* , 1929	*Απó τη Γη στον Άρη* , 1929
*To the unknown Gods* , 1930	*Στους άγνωστους θεούς* , 1930
*Days of terror* , 1932	*Μέρες Τρóμου* , 1932
*The iron gate* , 1925	*Η Σιδερένια Πóρτα* , 1925
*The house of the reptiles* , 1933	*Το σπίτι των Ερπετών* , 1933
*The Storms* , 1945	*Οι Τρικυμίες* , 1945

## CULTURAL AND HISTORICAL CONTEXT

To our knowledge, Voutyras is the only known Greek fiction writer who officially suffered from early-onset epileptic seizures. His prose narrative works are almost exclusively linked to the social realism framework, and pioneering the transition, in Modern Greek literature, from ethography to urban prose.

His world was that of the bourgeoisie and the urban working class. His imagery is reflective of nostalgic reminiscences of the poor quarters and suburbs of the city, and of the wretched life of its inhabitants. 4 The deprivation, the social disadvantage and relegation to the fringes of society, in which he was forced to live, the anguish of the man who desperately seeks work, the depressing and psychopathic atmosphere of poverty, relevantly portray the cultural and historical contexts. These topics were also approached by Maxim Gorky (1868–1936) and, in fact, Voutyras was profoundly influenced by Russian authors.

Voutyra's prose is characterized by precision in details blended with his perpetual imagination. leading to long descriptions (which are possibly hypergraphic due to his compulsively detailed accounts). This led to an anarchic style of writing rather than a structured work composition. Moreover, the psychological profiling of ordinary people seen in his writings, his dislike for social injustice and religious teachings, and, instead, his attraction for troubled atmospheres, set his technique apart as resistant to any esthetic analysis. 5

The subject that dominated his writings was the life of marginalized people in Piraeus. Their life and mindset (that is, their refusal to integrate an organized society) were portrayed in melancholic colors, of almost utopic vision. These depictions take on an almost symbolic meaning, 5 leading to a social reassessment of the early twentieth century, via the additional inclusion of elements of metaphysics and science fiction. This tendency was in part explained by Voutyras' ideological background, and possibly enhanced by his intensified emotion and depressive idiosyncrasy, frequently determined by (mostly non-dominant hemispheric) interictal temporal dynamics. 6 .

## NARRATIVE-BASED MEDICINE

During the past decades, many clinicians undertook the challenging task of making retrospective diagnoses of nervous and mental diseases. Accordingly, in the second half of the twentieth century, talented neurologists gathered literary depictions of epileptic phenomena, such as those seen in the fictional characters created by Fyodor Dostoevsky (1821–1881) and Miguel de Cervantes (1547–1616). Dostoevsky's epileptic seizures have extensively been linked to his extraordinary literary style, and they represent the prototype of such pathographic inquiry. Along with other artists of the early twentieth century, Voutyras helped deepen neurobehavioral knowledge on epileptology and on the concept of temporal lobe personality. Using horror imagery, social drama, fiction, satiric reconstructions, and gothic style (such as in the anthology “The ship of death and other tales”), he provided insight into the neuroesthetic dynamics of his epilepsy, probably TLE. This is consistent with previous studies assuming a right TLE diagnosis based on the works of writers, such as the eminent nineteenth century Brazilian litterateur Machado de Assis (1839–1908). 7

Dostoevsky's description of ecstatic auras received particular attention both from the general public and neurologists. Contradicting Gastaut (1978), 8 who reported on the rarity of the phenomenon described by Dostoevsky, Cirignotta et al. 9 were able to document the temporal origin of an ecstatic aura via the implementation of right temporal lobe ictal electroencephalography (EEG). These authors 9 showed for the first time that TLE can cause seizures that are subjectively experienced as “ecstatic” states. Thus, a particular emotional tone can be attributed to the use of the expression “ecstatic experience” regarding seizures that have an impact on personality and imagination.

In the present essay, we describe the role seizures played in Voutyras' novels and the potential use of such information to retrospectively advance some diagnostic hypotheses regarding his condition through his literary style and neuroesthetic reflections. We occasionally provide instances of supposed seizure-related quotes. Finally, an effort was made to interpret these quotes in the light of the complex interplay between his writing style and seizure activity as determined by his prodromal and/or early ictal (the “aura”) manifestations.

## TEMPORAL LOBE PERSONALITY

Chronic TLE causes mild interictal personality changes with a slowly but progressive cumulative effect. 10 This links to early evidence 11 12 pointing to the relationship involving TLE, specific interictal personality traits, and psychopathological symptoms known as Geschwind syndrome. 13 Waxman and Geschwind 11 focused on specific interictal behavioral phenomena, including hypergraphia, hyperreligiosity, and hyposexuality, circumstantiality, cyclothymia, intensified mental life, and odd social behavior. 14 Moreover, affective deregulation, irritability and impulsiveness, anxiety and obsessive-compulsive symptoms, paranoia, abnormal social interaction, grandiosity, schizophrenic-like symptoms and dissociative states have also been reported. 15 16

Hypergraphia, in particular, constitutes a symptom frequently observed in TLE. It is characterized by a strong desire toward compulsive writing, making detailed accounts of ordinary or personal life events or both, and writing poetry and fiction. It can even include lists or the mere repetition of written words, which are complex on a lexical and syntactic level. According to other authors, 17 18 the interictal behavioral manifestations of TLE do not represent a distinct nosological entity, but rather are conceptualized as separate interictal features or abnormalities. In many instances, hyperreligiosity may manifest as a deeply-felt atheism, 19 probably representing the two sides of the same coin. The “typical personality” of TLE patients, besides a deepening of emotionality, encompasses a salience-attribution tendency to ordinary events. In many instances this is expressed through their tendency to take on a cosmic view, that is, hyperreligiosity or intensely-professed atheism, 11 as seen in Voutyras's aversion for God and religious issues (attributed in part to his Marxist views as well).

Involvement of the right cerebral hemisphere has been suggested, which is in line with right temporal interictal discharges in ecstatic auras. 9 Hyperreligiosity and hypergraphia frequently occur in patients with TLE of the non-dominant hemisphere. 20 21 22 Instead, other authors suggest a bilateral involvement 14 or damage to the right hippocampus 21 in the manifestation of Geschwind syndrome in TLE.

High religiosity scores on the Bear–Fedio Inventory and morphological abnormalities in the temporal lobe may account for the association between religious delusions and postictal psychosis; 23 smaller hippocampal volumes in the right hemisphere have been reported in more religious patients compared with a nonreligious group. 21 Compared to other neurological conditions, TLE is remarkably associated with religiosity. Similarly, religious preoccupation has also been reported during the course of cases of dementia involving the frontal lobes. Edwards-Lee et al. 24 suggest that religiosity may primarily relate to right-frontal pathology, while the temporal lobes are also implicated.

## SOME EPISTEMOLOGICAL CONSIDERATIONS

The temporal lobe personality or *interictal personality disorder* has generated a great deal of controversy, 25 since its existence is taken as self-explanatory and unquestionable. 26 Instead, no mention is made in the current definition of epilepsy by the International League Against Epilepsy (ILAE), nor in current psychiatric diagnostic manuals, such as the Diagnostic and Statistical Manual of Mental Disorders, Fifth Edition (DSM-5) and the International Classification of Diseases, 10th revision (ICD-10). There has been scarce evidence of any specific association between these behavioral features and epilepsy, while the whole array of symptoms is rarely encountered in a single patient. Jasionis 26 calls attention to the clinical implications and proposed alternative explanations for the symptoms. Before proceeding further, we briefly discuss the logic underlying the present work as well as other inquiries on retrospective diagnosis.

Retrospective diagnosis has been criticized as an inappropriate method in medical history, and an interesting epistemic challenge against the retrospective diagnosis of historical figures points to the impossibility of scientific verification of a diagnosis in the past. 27 28 During premodern times until the nineteenth century, clinical diagnoses were exclusively made based on symptoms (syndromic) and with the intention to offer treatments, 29 30 while the concept of differential diagnosis (that is, distinguishing a disease from another or from a healthy status, or verifying against diagnostic standards) did not exist. Accordingly, it is contradictory and anachronistic to demand historicized diagnoses of historical figures while, at the same time, expecting non-historicized identification and verification in modern terms. 31 Muramoto 31 in his ontological and epistemic discussion of the topic proposed that conflating nosology with the act of diagnosing a patient is an error, since medical diagnosis is essentially a hypothesis-generation process and an explanatory device operating under various degrees of uncertainty and scarcity of information. Therefore, it is closer to a probabilistic (Bayesian) judgment with varying degrees of plausibility under uncertainty, rather than an apodictic judgment (true or false) as suggested by the critics. Retrospective diagnosis should be conceived not only from a medical point of view but also in the light of the wider spectrum of humanistic and social sciences by its overall plausibility and consistency.

For instance, syndromic diagnoses of functional disorders (such as fibromyalgia, chronic fatigue syndrome) and of many psychiatric disorders are made without any confirmatory test; yet, such diagnoses are highly practical accounts of the state of affairs of the patient in terms of treatment and prognosis. Of note, much of the criticism to such “anachronistic diagnoses” is decontextualized, since it often neglects the fact that different diseases might have coexisted in history, or that the same disease might have been described by means of different personal and clinical accounts, dictated by a particular historical context. Hapton e al. 32 suggested that clinicians could reach the correct diagnosis more than 80% of the time based on clinical history alone, without examining the patient or obtaining laboratory tests. These diagnoses are mostly syndromic and consistent with those advanced in retrospective diagnoses.

## WRITING ATMOSPHERE AND AURAS

Some narratives by Voutyras resemble paradoxology and the horror inherent in the writings of H. G. Wells and Edgar Allan Poe, 5 while Sachinis 33 considered Voutyras one of the most “demonic” personalities in modern Greek prose. “Dark conditions” are dominant features of his tales, such as the evocative presence of shadow, and the use of expressionistic elements, metaphysical suggestions, and characters suffering from psychopathological conditions. Voutyras's contribution to the psychographic narrative, similar to that of Strindberg and Dostoevsky, strives toward the search for the psychological determinants of social and individual behavior, the relationship between need and desire, and conscious and unconscious states.

Voutyras's elliptical narratives and continuous time shifts from the past to the present and to the future are comparable to those of Joyce, Sartre and Faulkner. Temporal lobe epilepsy may induce aberrant time processing, in which the present may be mistaken for the past, and the past, for the present; the real may be seen as unreal, and vice versa; time may be sped up or slowed down. 34 All these elements are present in the work of Voutyras.

For Voutyras, time acquires a new role: it ceases to be the documentary time and becomes a parameter of consciousness. There is a gradual contraction of time from one pair to another (such as, first night-first day, second night-second day) which intensifies the myth and the events in it. Narrative fluidity is also remarkable. Indeed, both narrative space and time constantly change, in an attempt to highlight the internal elements of the myth. At the same time, the writer is somewhat “indifferent” to their external organization.

[…the place: tavern – house – shop; the time: The night in the tavern, the six consecutive moments of time...].

Voutyras's style breaks the rules of ordinary spontaneity and structure of writing. Without predefined rules, he deliberately combines fantasy and reality,. He forms a purely personal narrative time, “playing” with what the reader's expectations with constant twists.

As Dimaras 5 suggests, Voutyras's “writing style weaknesses” (that is, telegraphic composition, delusions), as described by some critics, are typical features of the psychological realism stemming from the author's unconstrained spirit and his “primitive as well amateurish insightful sensitivity”. He did not pay much attention to language matters in his writing, and he was not interested in external literary features. 4 However, Voutyras's stories have an inner coherence, logical consistency, and genuine poetic sense. He uses strong images, metaphors, and shows rare observation skills. In particular, the metaphors and oxymora contained in his fictional works may constitute traces of seizures popping out during auras. 35

The relationship between epilepsy and literary metaphors, may be expressed by means of either *seizures and epilepsy used as metaphors* or *seizures described in metaphors* . 34 The former includes sexual metaphors; metaphors involving strong emotions, life crises and breakdowns, and also exultation; religious metaphors; and metaphors of weakness; while the latter is rarely reported by writers with epilepsy. 34

In fiction, both the above are likely to reflect societal stereotypes, while seizures are conceived as bordering on religion and sexuality, as expressions of critical emotions ending in ecstasy or breakdown. Approximations to the experience of a seizure typically refer to either dreams or intensive sensory experiences. Of note, the use of metaphors by writers suffering epilepsy and by those who possess knowledge on epilepsy differs only superficially. 34

Epileptic auras may represent a precious instrument in the way many litterateurs who are sufferers perceive their own selves and the world. In addition, the study of these experiential phenomena may promote understanding of new and enlightening aspects of epilepsy. The bizarreness and unusualness of aural and ictal subjective experiences are of such an “intensity” that patients may lack the expressive means to describe them. This is probably the reason why self-reports of epilepsy often are rich in metaphors and oxymora. 36

For instance, in his narrative “The city of the curse”, the following oxymoron can be seen: “and the child with the old face who came to be paid and he smelled like that [...]. But he was not a child, he was an old man with something childish!” 37 A metaphor possibly belonging to this kind of phenomena taken from his narrative “Place of souls” is as follows: “wild grass grew everywhere, high and thick, in the windows, on the walls, in the windows, which, like eyes outstretched, were skeletal eyes”. 37

Superstitions and feelings of prescience expressed by his characters – often encountered in seizures of temporal lobe origin – are key elements in his stories. In “The revenge that will come”, one reads: “one morning he got up with a premonition that something bad was going to happen. He had also seen a dream”. 37

The oneiric atmosphere of his prose makes the reader discover dream worlds through suggestion techniques. It is believed that Voutyras possessed a deep knowledge of the troubled mind, probably because of his own experience with epilepsy.

These stories abound with cemeteries, blood rites, murders, scary dreams, dim lights, premonitions, obsessions, and illusions, all impregnated with a deadly savagery, and possibly rooted in the artist's temporo-limbic epileptogenesis. Voutyras portrayed heroes surrender after fighting in vain the darkness: “With the one who secretly directs these blows, how was it possible for him to fight? This is hidden everywhere, in the light of day, in the dark, in the air!”. 37 It is a world of the living dead whose peers do not exist in Greek literature:

“The darkness, or its shadow, began to fall, and in it the black companions of the night began to fly fast […].”

“at that moment, along with it, a shadow, a dark figure of a man stood on the side of the road […]”

“the wind did not blow, and the giant heavy-headed pines, the tall cypresses remained motionless, black, dark […]”

“I was on a dark and mournful street in Piraeus […]”

“and the night came wild, dark as hell, and like the sword of an angel the lightning tore it every now and then […]”

“someone in the dark with an ax dug a tree trunk to make a baby crib […]” 37

Interestingly, indications of weakness and vulnerability are often met as prototypes of epilepsy metaphors. 34 For instance, in “The city of the curse”: “but also in the shops that were crowded, they spoke slowly, silently, tired, or they would keep a silence similar to the one that only death brings […]”. 37

## LITERATURE-BASED CLINICAL EVIDENCE

As Voutyras stated,

The first blow came to me in lower secondary school. I fell down during the lesson. Others tried in vain to help me recover. Where I was thinking something and somewhere my imagination was dragging me […] something was presented and with that I fell. This thing was presented as an old, forgotten memory. And it was so sweet that it led me for a while to a state of non-existence […].

In “The revenge that will come” a déjà vu experience becomes evident:

Paul listened carefully, but suddenly something circled him like a dream, or a piece taken from sleep, and it seemed to him then that what the teacher had said, the piece of Scripture and that he would still say, if he knew everything, everything, someone to have told him, to have read them to him on a card at other times, in mysterious hours! 37

Voutyras began his writing carrier at the age of 35 years. Τhat illness that had oppressed me so much, had stopped bothering me […]. It did not bother me anymore from the day I lost my way to music […]. After he did everything, so I can say, to throw me in the literature, he left me”. He studied to become an officer in the Greek Merchant Marine Academy, a profession that he never pursued because of his seizures; moreover, he practiced fencing, and later on took tenor lessons. Epilepsy, however, distanced him from a music and a career as a tenor. And, as he himself wrote, “like a hand or a kick, it pushed me and threw me into literature”. 38

This piece of autobiographic evidence leads us to hypothesize that the Greek writer suffered déjà vus linked to seizure activity of temporal lobe origin. Such a highly relevant clinical information, along with the general picture gathered through the idiosyncratic qualities emerging from his work, further support our literature-based hypothesis. In “The Prescience”, 39 the use of metaphors on strong emotional states such as fear, horror and prescience provides a unique insight into the writer's possible temporal auras.

“But I will tell you now, how wild animals, birds, vultures, cry, mourn when it is to fall in the place where they have lived for years, in their homeland, some great calamity! […] I rushed out, on the street. But as soon as I came out from the shadow of the house and was illuminated by the light of the moon, a different voice, wild with fear, was heard coming from nearby […]I raised the wood. I did not see anything. “But what is this!”, I said. Isn't it a night owl, owl? […] Isn1t it the owl of Athens, and it will be, and it cried for some great evil, great evils, that will fall in Athens and Greece? 39

## AN EVOLUTIONARY ACCOUNT

Mystical experiences are widely linked to temporal lobe function, 40 which is evolutionarily linked to the early stages of corticalization in humans, 41 as temporal structures initially emerged from the archaic, “demonic”, and bestial limbic “soul”. Newberg and d'Aquili 42 suggested that Dostoevsky's mystical ecstatic seizures might have produced the experience of mystical oneness, thus pointing to the supposedly underlying brain areas. Given the rarity of such epileptic phenomena, their etiological study has been difficult. In mystical experience, the role of the limbic system is well recognized, with the amygdala translating sensory inputs into emotions to produce a sense of religious fear. Once the limbic system is activated by the fight-or-flight responses or the rapid-eye-movement (REM) switch, the mystical feeling of unity may depend more on the limbic system per se and less on the brainstem that ignited it. 42

In “The revenge that will come”, an ecstatic aura can be observed:

Τhe teacher [Here, Voutyras means the political instructor speaking to the workers] was talking, talking, but he was no longer paying attention, he was immersed in that magician, who, like a cloud invisible to others, had surrounded him and made him feel so much, and as if he heard other voices saying to him. 37

In conclusion, we suggest that the psycholiterary profile of Voutyras is consistent with the idiosyncratic characteristics of the temporal lobe personality, while a non-dominant temporal lobe contribution has been proposed. One might indirectly discern a sort of resemblance of Voutyras epilepsy, abounding of ecstatic auras and déjà vus, mystical and dark elements and profound analytic detailing, with the aberrant interictal temporo-limbic dynamics (mostly arising from non-dominant temporal areas) encountered among renowned artists suffering from TLE and presenting with temporal lobe personality. The lack of composition, put forward as a criticism to his work, might be linked to the fundamental disruption of self-awareness induced by seizures. Moreover, several writers suffering from epilepsy have used aura experiences in their works, 34 thus producing an interictally “charged” literary atmosphere as that seen in Voutyras's narratives.

Finally, from a conventional scientific viewpoint, Voutyras's epilepsy type, like in the case of Dostoevsky, may easily turn “into a 'cul-de-sac' debate” 43 from a historical one; however, such retrospective diagnostic investigations may show the way literature may impact science through a deepening of the clinical understanding, and, under certain conditions, anticipating future discoveries as well.

Last but not least, detecting qualitative information on retrospective diagnoses from the literature may represent an indirect way of reviving the clinical acumen that once was of vital importance to clinicians. Those professionals were able to formulate diagnostic hypotheses and subordinate data to their clinical reasoning, “not the inverse, as passive pursuing of instrumental data makes clinical reasoning follow instrumental data as a slave follows his master”, as Luria advised. 44

## References

[1] Vitti M History of Modern Greek Literature Athens Ulysses 2008

[2] Dimosthénis Voutyrás (1872–1958) - Auteur - Ressources de la Bibliothèque nationale de Francehttps://data.bnf.fr/ark:/12148/cb13491921t

[3] Valsa M“Le mouvement intellectuel grec moderne”, La Revue de l' Époque, 11, 1920

[4] Politēs L A history of Modern Greek literature Oxford Clarendon Press 1973

[5] Dimaras CTh History of Modern Greek LiteratureState Univ. New York P., n.d.

[6] Bear D M Fedio P Quantitative analysis of interictal behavior in temporal lobe epilepsy Arch Neurol 1977 34 08 454 467 10.1001/archneur.1977.00500200014003 889477

[7] Guerreiro C A Machado de Assis's epilepsy Arq Neuropsiquiatr 1992 50 03 378 382 10.1590/s0004-282x1992000300020 1308419

[8] Gastaut H Fyodor Mikhailovitch Dostoevsky's involuntary contribution to the sympotomatology and prognosis of epilepsy. William G. Lennox Lecture, 1977 Epilepsia 1978 19 02 186 201 10.1111/j.1528-1157.1978.tb05030.x 346348

[9] Cirignotta F Todesco C V Lugaresi E Temporal lobe epilepsy with ecstatic seizures (so-called Dostoevsky epilepsy) Epilepsia 1980 21 06 705 710 10.1111/j.1528-1157.1980.tb04324.x 7439135

[10] Devinsky J Schachter S Norman Geschwind's contribution to the understanding of behavioral changes in temporal lobe epilepsy: the February 1974 lecture Epilepsy Behav 2009 15 04 417 424 10.1016/j.yebeh.2009.06.006 19640791

[11] Waxman S G Geschwind N Hypergraphia in temporal lobe epilepsy Neurology 1974 24 07 629 636 10.1212/wnl.24.7.629 4209727

[12] Waxman S G Geschwind N The interictal behavior syndrome of temporal lobe epilepsy Arch Gen Psychiatry 1975 32 12 1580 1586 10.1001/archpsyc.1975.01760300118011 1200777

[13] Geschwind N Behavioural changes in temporal lobe epilepsy Psychol Med 1979 9 02 217 219 10.1017/s0033291700030713 472070

[14] van Elst L T Krishnamoorthy E S Bäumer D Psychopathological profile in patients with severe bilateral hippocampal atrophy and temporal lobe epilepsy: evidence in support of the Geschwind syndrome? Epilepsy Behav 2003 4 03 291 297 10.1016/s1525-5050(03)00084-2 12791331

[15] Bear D Freeman R Greenberg M Behavioral alterations in patients with temporal lobe epilepsy. Psychiatric aspects of temporal lobe epilepsy Washington American Psychiatric Association Press 1984;11:198340468–9.

[16] Blumer D Evidence supporting the temporal lobe epilepsy personality syndrome Neurology 1999 53(5, Suppl 2)S9 S12 10496229

[17] Mungas D Interictal behavior abnormality in temporal lobe epilepsy. A specific syndrome or nonspecific psychopathology? Arch Gen Psychiatry 1982 39 01 108 111 10.1001/archpsyc.1982.04290010080014 6948536

[18] Dodrill C B Batzel L W Assessment of psychosocial and emotional factors in epilepsy The Neurobehavioral Treatment Epilepsy 1993:265–83

[19] Heilman K M Valenstein E E Clinical neuropsychology Oxford University Press 2003 10.1016/j.clinph.2003.10.027

[20] Roberts J K Robertson M M Trimble M R The lateralising significance of hypergraphia in temporal lobe epilepsy J Neurol Neurosurg Psychiatry 1982 45 02 131 138 10.1136/jnnp.45.2.131 7069424 PMC1083040

[21] Wuerfel J Krishnamoorthy E S Brown R J Religiosity is associated with hippocampal but not amygdala volumes in patients with refractory epilepsy J Neurol Neurosurg Psychiatry 2004 75 04 640 642 10.1136/jnnp.2003.06973 15026516 PMC1739034

[22] Okamura T Fukai M Yamadori A Hidari M Asaba H Sakai T A clinical study of hypergraphia in epilepsy J Neurol Neurosurg Psychiatry 1993 56 05 556 559 10.1136/jnnp.56.5.556 8505651 PMC1015019

[23] Kanemoto K Kawasaki J Kawai I Postictal psychosis: a comparison with acute interictal and chronic psychoses Epilepsia 1996 37 06 551 556 8641232 10.1111/j.1528-1157.1996.tb00608.x

[24] Edwards-Lee T Miller B L Benson D F The temporal variant of frontotemporal dementia Brain 1997 120(Pt 6):1027 1040 9217686 10.1093/brain/120.6.1027

[25] Benson D F The Geschwind syndrome Adv Neurol 1991 55 411 421 2003418

[26] Jasionis A „Epilepsinės asmenybės“ sindromo Kritika Criticism of “epileptic personality” syndrome Neurologijos Seminarai 2019 23 81 176 180

[27] Foxhall K Making modern migraine medieval: men of science, Hildegard of Bingen and the life of a retrospective diagnosis Med Hist 2014 58 03 354 374 25045179 10.1017/mdh.2014.28 PMC4103393

[28] Karenberg A Moog F P Next emperor, please! No end to retrospective diagnostics J Hist Neurosci 2004 13 02 143 149, discussion 166–167 15370320 10.1080/0964704049052158

[29] Shorter E The history of the doctor-patient relationship. Companion Encyclopedia of the History of MedicineEdited by: Bynum WF, Porter R.New York Routledge 1997 783 800

[30] Nicolson M The art of diagnosis: medicine and the five senses. Companion Encyclopedia of the History of MedicineEdited by: Bynum WF, Porter R.New York Routledge 1997 801 825

[31] Muramoto O Retrospective diagnosis of a famous historical figure: ontological, epistemic, and ethical considerations Philos Ethics Humanit Med 2014 9 01 10 24884777 10.1186/1747-5341-9-10 PMC4049481

[32] Hampton J R Harrison M JG Mitchell J RA Prichard J S Seymour C Relative contributions of history-taking, physical examination, and laboratory investigation to diagnosis and management of medical outpatients BMJ 1975 2(5969):486 489 10.1136/bmj.2.5969.486 1148666 PMC1673456

[33] Sachinis A Postwar and Interwar prosaists Thessaloniki Estia 1979(In Greek).

[34] Wolf P Epilepsy and metaphors in literature Epilepsy Behav 2016 57(Pt B):243 246 10.1016/j.yebeh.2012.04.067 26936537

[35] Wolf P The epileptic aura in literature: aesthetic and philosophical dimensions. An essay Epilepsia 2013 54 03 415 424 10.1111/epi.12051 23294431

[36] Surmann V Anfallsbilder: Metaphorische Konzepte im Sprechen anfallskranker Menschen Königshausen & Neumann 2005

[37] Dimosthénis Voutyras The ship of death and other tales. A narrative anthologyEdited by V. Tagkopoulos, Topos Editor,2011

[38] Voutyras DAutobiography. Journal Nea Estia 641958

[39] Voutyras D Twenty narratives Athens Dimitrakos 1924(In Greek).

[40] Saver J L Rabin J The neural substrates of religious experience The Neuropsychiatry Limbic Subcortical Disorders 1997 195 207 10.1176/jnp.9.3.498 9276850

[41] MacLean P D The triune brain in evolution: Role in paleocerebral functions Springer Science & Business Media 1990 10.1126/science.250.4978.303-a 17797318

[42] Newberg A d'Aquili E G Why God won't go away: Brain science and the biology of belief Ballantine Books 2008

[43] Iniesta I Dostoevsky's epilepsy: A contemporary “paleodiagnosis” Seizure 2007 16 03 283 285 10.1016/j.seizure.2006.11.003 17178237

[44] Cole M Levitin K Luria A The autobiography of Alexander Luria: A dialogue with the making of mind Psychology Press 2014

